# Assessment of infiltrative margins in subcutaneous soft-tissue sarcomas using ultrasonography and gadolinium-enhanced fat-suppressed T1-weighted imaging

**DOI:** 10.1016/j.sopen.2026.07.001

**Published:** 2026-07-13

**Authors:** Jun Iwatsu, Munenori Watanuki, Shinichirou Yoshida, Hirotaka Kurata, Shin Hitachi, Sota Oguro, Mika Watanabe, Toshimi Aizawa

**Affiliations:** a Tohoku University Graduate School of Medicine, Department of Orthopedic Surgery, 1-1 Seiryomachi, Aoba-ku, Sendai, Miyagi, 980-8574, Japan; b JR Sendai Hospital, Department of Orthopedic Surgery, 1-1-5 Itsutsubashi, Aoba-ku, Sendai, Miyagi, 980-8508, Japan; c Takeda General Hospital, Department of Diagnostic Radiology, 3-27 Yamagamachi, Aizuwakamatsu, Fukushima, 965-8585, Japan; d Tohoku University Hospital, Department of Diagnostic Radiology, 1-1 Seiryomachi, Aoba-ku, Sendai, Miyagi, 980-8574, Japan; e Tohoku Kosai Hospital, Department of Pathology, 2-3-11 Kokubuncho, Aoba-ku, Sendai, Miyagi, 980-0803, Japan

**Keywords:** Soft tissue sarcoma, Infiltration, Ultrasonography, Preoperative assessment, Magnetic resonance imaging

## Abstract

Magnetic resonance imaging (MRI) aids in detecting infiltrative growth; however, preoperative determination of the extent of soft-tissue sarcoma (STS) infiltration remains challenging. Ultrasonography could also detect infiltration effectively. We investigated the usefulness of MRI and ultrasonography as preoperative assessments for infiltrative STS. We retrospectively evaluated the data of 24 patients with subcutaneous infiltrative STS. Infiltrative length was measured using gadolinium-enhanced fat-suppressed T1-weighted imaging (GdFsT1) (M-IL) and ultrasonography (U-IL). The reactive layer was set at a greater distance of M-IL or U-IL. The surgical margin was set at greater distance of 2 cm from the tumor edge or 1.5 cm beyond the reactive layer. Histological infiltrative length (H-IL) and histological surgical margin length (H-margin) were measured using specimens. Relationships among M-IL, U-IL, and H-IL were analyzed using Spearman's rank correlation coefficients. Clinical outcomes, such as overall survival (OS) and local recurrence (LR), were evaluated using the Kaplan–Meier method. In 19 of 64 directions, H-IL was wider than M-IL. Among the 19 directions, U-IL was wider than H-IL in 11 directions, whereas H-IL was wider than U-IL in 8 directions. Mean H-margin was 29 mm, and the margin was negative in all directions. Spearman's correlation revealed weak associations between H-IL and M-IL (ρ = 0.08, *p* > 0.05) and H-IL and U-IL (ρ = 0.16, p > 0.05). The 5-year LR and OS were 91% and 87.5%, respectively. GdFsT1 alone underestimated STS infiltration. Combined MRI and ultrasonography represent a feasible approach for preoperative assessment and aids in achieving margin-negative resection for subcutaneous STS.

## Highlights

• In 30% of directions, H-IL was wider than M-IL.
• Of these directions, U-IL was wider than H-IL in 58% of directions.
• Histological surgical margins were negative in all directions.
• There were weak associations between H-IL and M-IL and between H-IL and U-IL
• The 5-year local recurrence-free and overall survival rates were 91% and 87.5%

## Background

Soft-tissue sarcomas (STS), such as undifferentiated pleomorphic sarcoma (UPS) and myxofibrosarcoma (MFS), can exhibit an infiltrative growth pattern [1], [2]. These tumors and the infiltrative areas require wide resection to prevent local recurrence, which is significantly associated with a poor prognosis [3], [4]. Magnetic resonance imaging (MRI) indicates infiltrative growth as a tail-like, extensive lesion [5], [6]. Gadolinium (Gd)-enhanced MRI is a common approach for determining the infiltrative area of STS [6], guiding a surgical plan. However, determining the infiltrative area preoperatively and selecting the accurate imaging are challenging [7].

Ultrasonography has potential as a first-line examination for STS because it is simple and minimally invasive [8], [9], [10]. In addition, the localized fascial thickening of infiltrative sarcomas can be detected using preoperative ultrasound [1], [11]. Oguro et al. have reported the potential of contrast-enhanced ultrasonography in evaluating the extent of subcutaneous STS [12]. These studies demonstrated the utility of ultrasonography in determining STS infiltration, guiding surgical plans. Nevertheless, reports on the combination of ultrasound and MRI to determine the surgical plan are limited. Moreover, comparisons of the imaging and histological results are lacking.

In this study, we aimed to evaluate the association between the infiltrative area of subcutaneous STS assessed using MRI, ultrasonography, and histopathology and assess patient outcomes.

## Methods

This retrospective study was conducted per the ethical standards of the Declaration of Helsinki and approved by the Institutional Review Board of Tohoku University Hospital (approval number: 2024-1-153). The requirement for informed consent was waived. The eligibility criteria were the detection of subcutaneous infiltrative STS using MRI and availability of preoperative ultrasonography conducted between 2010 and 2013 in a single institution; absence of preoperative radiotherapy; and availability of sufficient clinical information.

**Fig. 1:**
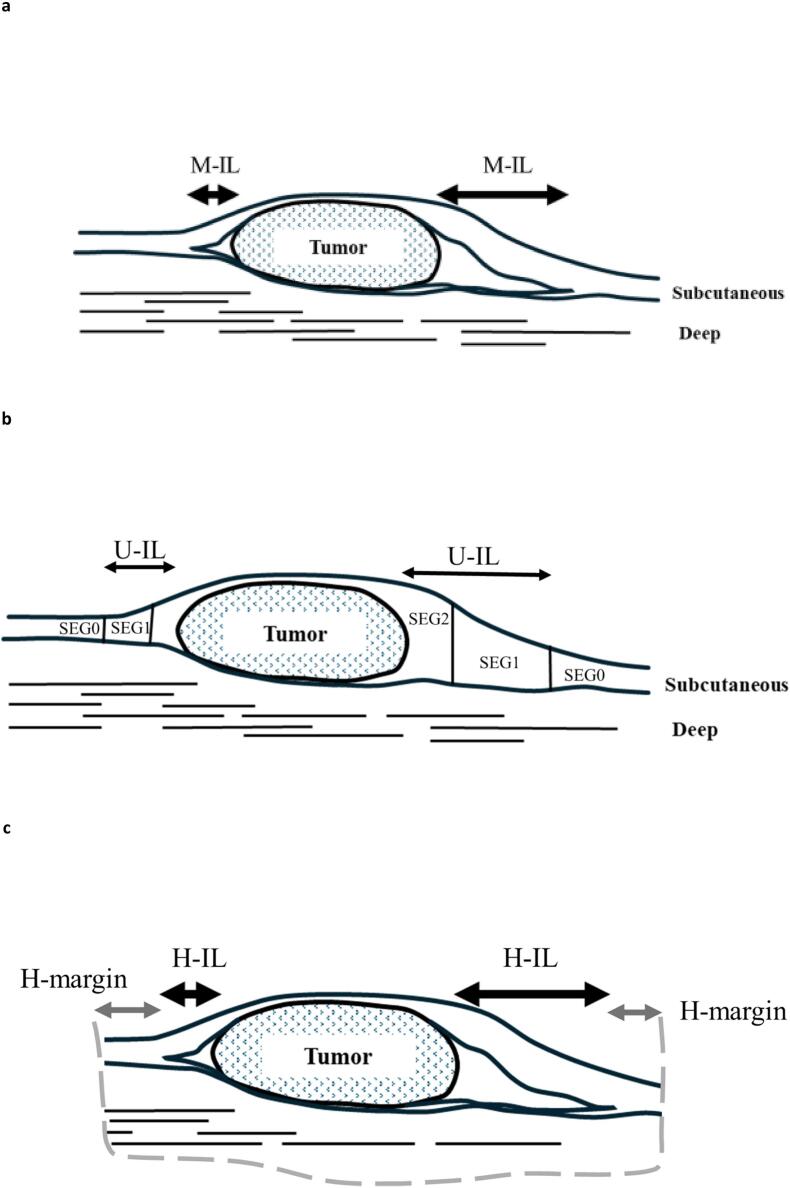
Scheme depicting the infiltrative length and the histological surgical margin. (a) Black double-headed arrow shows soft tissue sarcoma (STS) infiltration evaluated using gadolinium-enhanced fat-suppressed T1-weighted imaging (GdFsT1) (M-IL). (b) Black double-headed arrow shows STS infiltration evaluated using ultrasonography (U-IL). Subcutaneous Echogenicity Grade (SEG): the boundary between SEG 0 and SEG 1 was defined as the outer edge of the reactive layer of tumors. (c) Histological infiltration (black double-headed arrow) of the specimens was evaluated by a pathologist postoperatively (H-IL). The grey double-headed arrow shows the histological surgical margin (H-margin).

MRI was performed 1 month preoperatively. For each tumor, the distance from the tumor surface to the outer edge of the infiltrative length was measured using gadolinium-enhanced fat-suppressed T1-weighted imaging (GdFsT1) in four directions (0, 3, 6, and 9 o'clock directions) (M-IL). This approach was based on a previous study, which suggested that the M-IL correlated with the histological infiltrative area (H-IL) [13] (Fig. 1a). Ultrasonography was performed 1–2 days preoperatively. The subcutaneous tissue surrounding the tumor was evaluated using the Subcutaneous Echogenicity Grade (SEG); the boundary between SEG 0 (normal) and SEG 1 (moderate) [14] was defined as the outer edge of the reactive layer of the tumor, as tumor cells were found in the peritumoral edema [15] (Fig. 1b). The distance from the tumor edge to this boundary was measured in the same four directions (0, 3, 6, and 9 o'clock directions) using ultrasonography (U-IL).

**Fig. 2:**
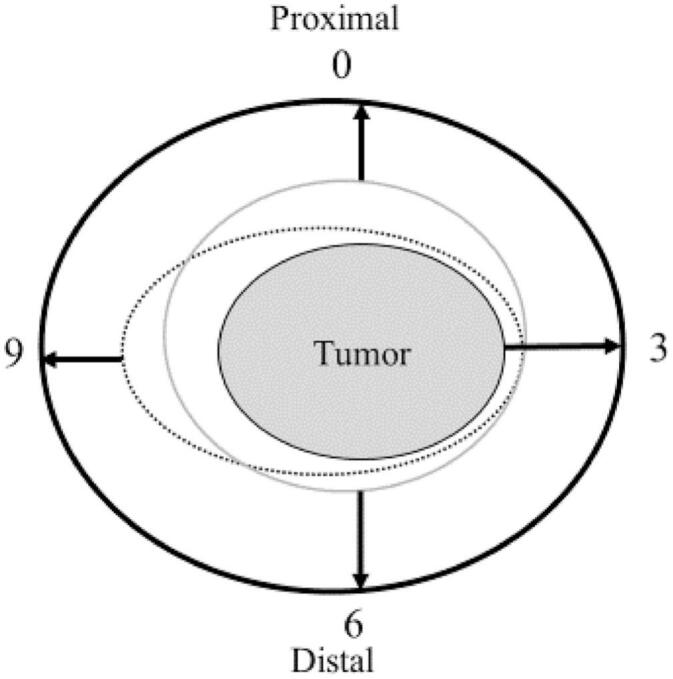
Soft-tissue sarcoma (STS) infiltration was evaluated using gadolinium-enhanced fat-suppressed T1-weighted imaging (GdFST1) and ultrasonography. The illustration depicts the grey line evaluated using ultrasound and the black dotted line evaluated using GdFsT1. The surgical margin was set as the greater distance of 2 cm from the tumor edge (3 o'clock direction) or 1.5 cm beyond the wider margin (0, 6, or 9 o'clock direction).

We determined the surgical plan based on the M-IL and U-IL. The reactive layer was set at a greater distance of M-IL or U-IL. The surgical margin was set at greater distance of 2 cm from the tumor edge or 1.5 cm beyond the reactive layer (Fig. 2). For preoperative diagnosis, a core needle or incisional biopsy was performed by orthopedic surgeons. An experienced pathologist diagnosed all specimens. A pathologist mapped the edge of the infiltrative area and measured H-IL and histological surgical margin length (H-margin), which was defined as the distance from the edge of the infiltrative area to the edge of the specimen.

Patient information, such as age, sex, tumor diameter, tumor site, pathological diagnosis, histological grade according to the Federation Nationale des Centres de Lutte Contre le Cancer (FNCLCC) grading system, postoperative radiotherapy, chemotherapy, and prognosis, was retrospectively obtained. Local recurrence was defined as the period between the surgery and local recurrence dates. When patients underwent additional excision after an unplanned excision, the surgery date was defined as the date of additional excision at our hospital.

### Statistical analysis

Spearman's rank correlation coefficient was used to investigate the correlations between M-IL, U-IL, and H-IL. Overall survival (OS) and local recurrence-free survival (LRFS) were defined as the time from initial surgery to death from any cause or last follow-up and local recurrence, respectively. Survival curves were generated using the Kaplan–Meier method. Data were analyzed using SPSS Version 29.0 (SPSS Japan Inc., Tokyo, Japan).

## Results

In total, 24 patients were included in this study. The demographics and clinical information are presented in Table 1. The median age of the patients was 71 (48–87) years. The pathological diagnoses were MFS (13 patients), UPS (6 patients), and malignant peripheral nerve sheath tumor (five patients). The tumors were in the forearm, lower limb, trunk, and thigh in 1, 3, 6, and 14 patients, respectively. All tumors were in the subcutaneous layer, with two infiltrating the deep layer. The maximum tumor diameter was 5.8 (3.0–10.2) cm. All patients, except one who had a residual tumor after an unplanned excision, underwent initial definitive surgeries at our institution. No patients underwent radiotherapy with a negative margin, except for one who underwent postoperative radiotherapy because of a positive margin in the deep layer. Only one patient received neoadjuvant chemotherapy. The FNCLCC grades were 3, 2, and 1 in 9, 12, and 3 patients, respectively. Regarding patient outcomes, two patients who did not receive radiotherapy showed local recurrence during follow-up. There were six metastasis cases, three of which led to death.

**Table 1:** Demographics and clinical information.

	*N* = 24
Age, years (mean)	48–87 (71)
Sex	
Male	16
Female	8
Follow-up period, months (mean)	12–74 (31)
Diagnosis	
UPS	6
MFS	13
MPNST	5
Location of tumor	
Thigh	14
Trunk	6
Lower limb	3
Forearm	1
Tumor diameter	3.0–10.2 (5.8)
Surgery	
Initial	23
After unplanned excision	1
Perioperative chemotherapy	
Yes	1
No	23
Postoperative radiotherapy	
Yes	1
No	23
FNCLCC	
1	3
2	12
3	9
Histological margin	
Negative	23
Positive	1
Status at last follow-up	
CDF	17
NED	1
AWD	2
DOD	3

CDF, continuous disease-free; NED, No evidence of disease; AWD, alive with disease; DOD, dead of disease; UPS, undifferentiated pleomorphic sarcoma; MFS, myxofibrosarcoma; MPNST, Malignant peripheral nerve sheath tumor; FNCLCC, Federation Nationale des Centres de Lutte Contre le Cancer.

**Fig. 3:**
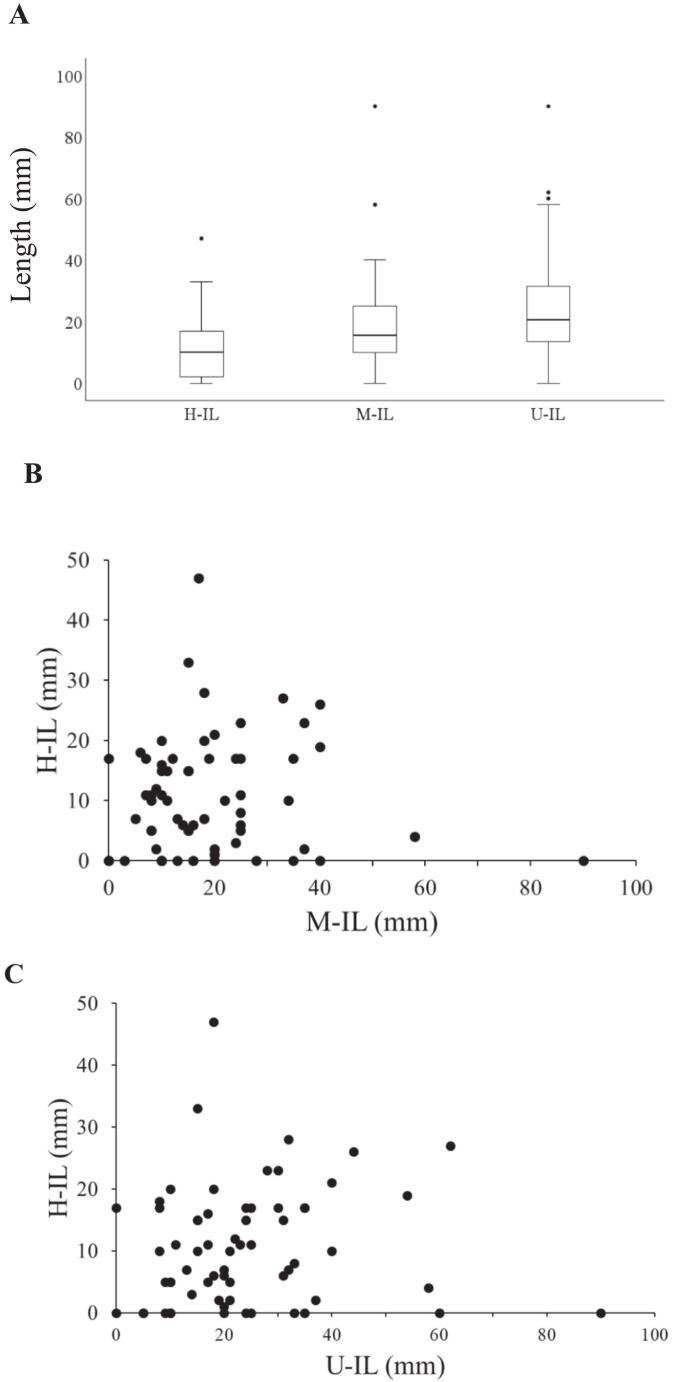
Box plot. (a) Box plot indicating the length distribution of the histological infiltration (H-IL), radiological infiltration determined via gadolinium-enhanced magnetic resonance imaging (GdFsT1; M-IL), and ultrasonographic infiltration (U-IL) demonstrating that the distributions of the U-IL and M-IL are overestimated compared with the H-IL. Relationship between the H-IL and M-IL (b) or U-IL (c). H-IL is more strongly correlated with the U-IL than with the M-IL.

Of 96 directions of the 24 tumors, 64 were analyzed to determine the relationship among M-IL, U-IL, and H-IL; the H-IL could not be measured in other directions because the specimens were not evaluated. The mean H-IL, M-IL, and U-IL were 11 (0–47), 19 (0–90), and 23 (0–90) mm, respectively (Fig. 3a). The H-IL was shorter than the M-IL and U-IL in 45 of the 64 (70%) directions. The H-IL was wider than the M-IL in 19 directions (30%). The H-IL was shorter than the U-IL in 11 of the 19 directions; however, the H-IL was wider than the U-IL in 8 directions (Table 2). In eight directions, the H-IL was on average 13 (2–30) and 12 (2–29) mm wider than the M-IL and U-IL, respectively; these cases achieved negative surgical margins by wide resection. In 12 directions, the M-IL and U-IL were >1 mm, whereas the H-IL was 0 mm. In one case, the H-IL was 17 mm, whereas the M-IL and U-IL were 0 mm (Fig. 3b and c). The correlations between the H-IL and M-IL and between the H-IL and U-IL were 0.08 and 0.16, respectively (*p* > 0.05).

**Table 2:** Relationship between histological infiltration (H-IL), ultrasonographic infiltration (U-IL), and radiological infiltration by magnetic resonance imaging (M-IL).

H-IL	>U-IL	≤U-IL	Total
>M-IL	8	11	19
≤M-IL	0	45	45
Total	8	56	64

The postoperative surgical margin in the horizontal direction was negative in all cases. The average H-margin was 29 (7–93) mm. The postoperative surgical margins were 0–10, 10–20, and >20 mm in 1, 18, and 45 directions, respectively (Table 3). The postoperative surgical margin in the deep layer was positive in one case.

**Table 3:** Histological surgical margin width (H-margin) in 64 directions.

H-margin	Number of directions
0–10 mm	1
11–20 mm	18
>20 mm	45

**Fig. 4:**
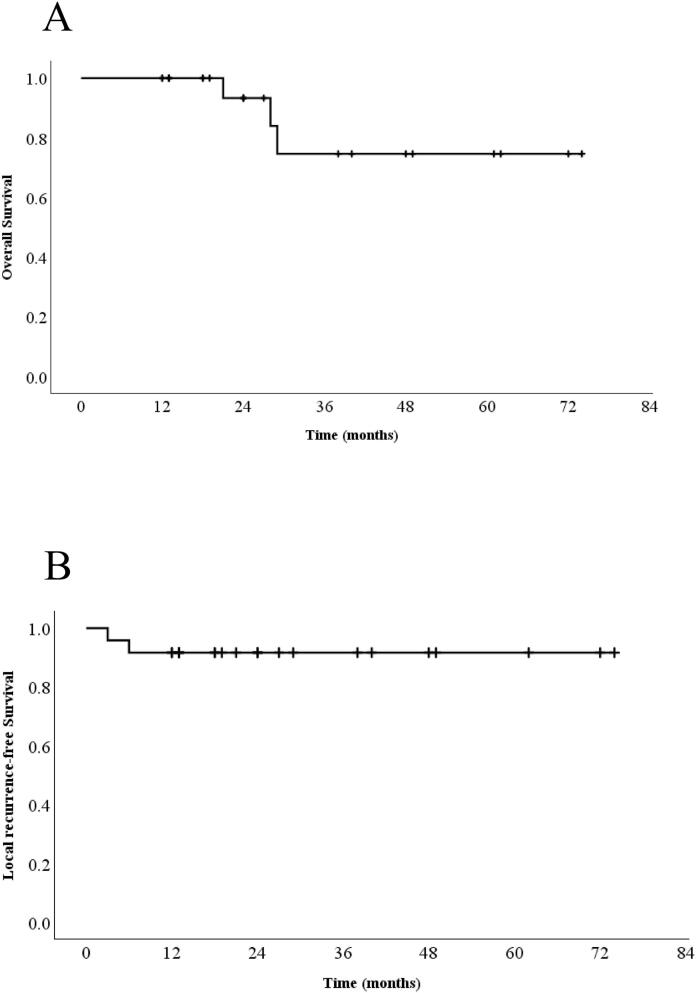
Kaplan–Meier curve. Overall survival (a) and local recurrence-free survival (b).

Concerning patient outcome, 17 of the 24 patients remained disease-free during follow-up. Two patients experienced local recurrence at 3 and 6 months postoperatively; six experienced lung or lymph node metastasis, and three died of the disease. The Kaplan–Meier curve showed that the 5-year LRFS was 91%, and the 5-year OS was 87.5% (Fig. 4).

## Discussion

To our knowledge, no previous study has assessed STS infiltrative areas using preoperative MRI, ultrasonography, and postoperative histopathology. In this study, we assessed infiltrative area of subcutaneous STS using MRI, ultrasonography, and histopathology and found that such evaluation using GdFsT1 sometimes underestimated the histological infiltration of the STS. Ultrasonography aided in determining the surgical plan for subcutaneous STS with a negative margin.

Infiltrative sarcomas exhibit a high risk of positive histological margins, which cause local recurrence [16], [17]. Liu et al. reported that a <10-mm margin was significantly associated with local recurrence, distant metastasis-free survival, and disease-specific survival [18]. In their assessment of sarcoma infiltration, Iwata et al. reported that radiological infiltration detected using GdFsT1 was significantly correlated with the histological infiltration of infiltrative STS [13]. However, it is challenging to accurately detect the infiltrative area to achieve a negative margin, as histological infiltration has been reported even when MRI indicated no infiltrative areas [13], [19], [20]. For example, Kaya et al. reported that 4 of 17 MFS without radiological infiltrations showed histological infiltrations [19]. In the present study, the H-IL was wider than the M-IL in 19 (30%) of the 64 directions, consistent with previous findings. This finding implies that GsFST1-based evaluation may underestimate STS infiltration.

Ultrasonography is simple, cost-effective, and requires no medications [21]. The infiltrative area can be evaluated in any direction under ultrasonography, whereas MRI enables measurements in only four directions: longitudinal and horizontal. In this study, 11 of 19 directions where the infiltrative area was confirmed to be longer than that indicated by MRI were covered by ultrasonography, suggesting the significance of combining MRI and ultrasonography in determining infiltrative areas. White et al. reported that tumor cells were found in peritumoral edema [15]. Ultrasonography detected the edema as tumor infiltration, and the edema was evaluated using the SEG. Nevertheless, in eight directions, the H-IL was 13 and 12 mm wider than the M-IL and U-IL, respectively. We planned a wide resection 1.5 cm beyond the infiltrative area, which achieved a negative margin in eight cases. As such, it is crucial to resect a wide margin from the edge of the infiltrative area detected using MRI or ultrasonography to prevent positive margins [17].

MRI and ultrasonography may detect swelling and inflammation around the tumor as an infiltrative area, which could cause overtreatment. Iwata et al. reported that 20% of 59 tumors with radiological infiltrations did not show histological infiltrations [13]. In the present study, 12 directions (19%) on GdFsT1 and ultrasonography with infiltrative findings showed no histological infiltrations. Radiological infiltration without histological infiltration might indicate edema or inflammation without tumors. Consequently, the surgical margin was larger than those reported in other studies [17]. All patients, except one with a positive margin in the deep layer, achieved a negative margin. Although the correlation among the M-IL, U-IL, and H-IL was weak, and MRI and ultrasonography tended to overestimate or underestimate STS infiltration, this finding describes the limitations of preoperative imaging and the significance of combining multiple modalities to achieve margin-negative resections. Ultrasonography tends to have a stronger correlation with the actual invasion distance (and is less likely to underestimate it) than MRI. This finding suggests that combined MRI and ultrasound is more reliable than MRI alone for safe surgical planning, as it reduces the risk of positive margins.

This study has some limitations. First, the specimen was shrunken; therefore, the pathological distance may have been underestimated. Second, this study had a small sample size and included only superficial cases, not deep or retroperitoneal cases. Third, only one surgeon conducted the MRI and ultrasonography, which were performed only once, and inter- and intra-rater reliability could not be determined. Finally, two cases with negative margins were recurrences. Kainhofer et al. reported that 12% of tumors had R0 margin recurrence within 5 years postoperatively [22], implying positive margins in some directions that were not evaluated in the stump.

## Conclusions

GdFsT1 alone may underestimate STS infiltration. The preoperative assessment of STS infiltration using ultrasonography combined with Gd-enhanced MRI could help achieve margin-negative surgeries. However, the preoperative accuracy of this assessment requires improvement.

## CRediT authorship contribution statement

**Jun Iwatsu:** Writing – original draft, Funding acquisition, Formal analysis. **Munenori Watanuki:** Writing – review & editing, Project administration, Conceptualization. **Shinichirou Yoshida:** Writing – review & editing, Project administration. **Hirotaka Kurata:** Investigation. **Shin Hitachi:** Investigation. **Sota Oguro:** Investigation. **Mika Watanabe:** Investigation. **Toshimi Aizawa:** Writing – review & editing.

## Ethics approval

This retrospective study was conducted per the ethical standards of the Declaration of Helsinki and approved by the Institutional Review Board of Tohoku University Hospital (approval number: 2024-1-153).

## Funding

This work did not receive support from any funding agency.

## Declaration of competing interest

The authors declare that they have no competing interests.
